# Assembly of a micro-hotspot of caenogastropod endemism in the southern Nevada desert, with a description of a new species of *Tryonia* (Truncatelloidea, Cochliopidae)

**DOI:** 10.3897/zookeys.492.9246

**Published:** 2015-03-30

**Authors:** Robert Hershler, Hsiu-Ping Liu, Jeffrey S. Simpson

**Affiliations:** 1 Department of Invertebrate Zoology, Smithsonian Institution, P.O. Box 37012, Washington, DC 20013-7012, USA; 2 Department of Biology, Metropolitan State University of Denver, Denver, CO 80217, USA

**Keywords:** Gastropoda, Assimineidae, Hydrobiidae, western United States, aquatic snails, biogeography, taxonomy, conservation

## Abstract

Newly obtained and previously published sequences of the cytochrome *c* oxidase subunit I (COI) gene were analyzed to examine the biogeographic assembly of the caenogastropod fauna (belonging to the families Assimineidae, Cochliopidae, and Hydrobiidae) of an isolated spring along the lower Colorado River in southern Nevada (Blue Point Spring). Based on available COI clock calibrations, the three lineages that comprise this fauna are 2.78–1.42 million years old, which is roughly coeval or slightly younger than the age of Blue Point Spring (inferred from local fossil spring deposits). Two of the lineages—endemic *Pyrgulopsis coloradensis* and Assiminea aff. infima—are most closely related to snails in the Death Valley area (well to the west) and likely colonized Blue Point Spring by transport on birds. A single haplotype was detected in both of these snails, suggesting that they may have only recently colonized Blue Point Spring. The third lineage—endemic *Tryonia infernalis*, newly described herein based on morphological and molecular evidence—is most closely related to a geographically proximal species in a lower Colorado River tributary (*Tryonia clathrata*); the split between these taxa may be the product of vicariance (severance of a prior drainage connection) or a separate jump dispersal event. The considerable genetic diversity in *Tryonia infernalis* (three haplotypes differing by 0.6% mean sequence divergence) suggests a possibly lengthy history of local differentiation. Our findings also identify Blue Point Spring as a new micro-hotspot of groundwater-dependent biodiversity in Nevada and will assist ongoing efforts to protect and conserve these imperiled ecosystems.

## Introduction

The desert region of southeastern California and southwestern Nevada, encompassing portions of the Great Basin and lower Colorado River watershed, contains distinctive assemblages of tiny caenogastropods—belonging to the families Assimineidae (genus *Assiminea*), Cochliopidae (*Tryonia*) and Hydrobiidae (*Pyrgulopsis*)—that have been a recent focus of biogeographic study using mtDNA sequence data (e.g., Hershler et al. 1999a, b, Hershler and Liu 2008a, b). These assemblages broadly overlap geographically and are tightly linked with spring habitats; their biogeographic histories do not well correlate with surface drainage and have likely been shaped, at least in part, by overland dispersal on waterfowl (e.g., Liu et al. 2003, Hershler et al. 2005, Liu and Hershler 2007, Hershler and Liu 2008a). The biogeographic patterns of these assemblages also differ in important respects. The regional assimineids (referred to herein as the *Assiminea infima* complex) are amphibious animals that typically live on riparian vegetation along the margins of springs and spring runs. This assemblage belongs to a single lineage that diverged from marine (Pacific) coastal progenitors during the late Pliocene (Hershler and Liu 2008a). The other two assemblages are entirely aquatic: *Tryonia* is restricted to thermal waters while *Pyrgulopsis* lives in ambient temperature and thermal habitats. Both of these assemblages are composed of multiple lineages, some having long histories of diversification within the region (Hershler et al. 1999a, Hershler et al. 2011). The *Tryonia* assemblage is composed of a few subgroups that have close relationships with congeners from the lower Colorado River basin (*Tryonia angulata* Hershler), northern Great Basin and western California (*Tryonia margae* Hershler, *Tryonia salina* Hershler), and northeastern Mexico (*Tryonia porrecta* [Mighels, 1845]; clade composed of *Tryonia elata* Hershler, *Tryonia ericae* Hershler, *Tryonia variegata* Hershler) (Hershler et al. 1999a, Hershler et al. 2011). The *Pyrgulopsis* assemblage contains a much larger number of lineages which have close relationships to taxa from western California, the lower Colorado River basin, and other portions of western North America (Hershler and Liu 2008b, Hershler et al. 2013).

**Figure 1.**
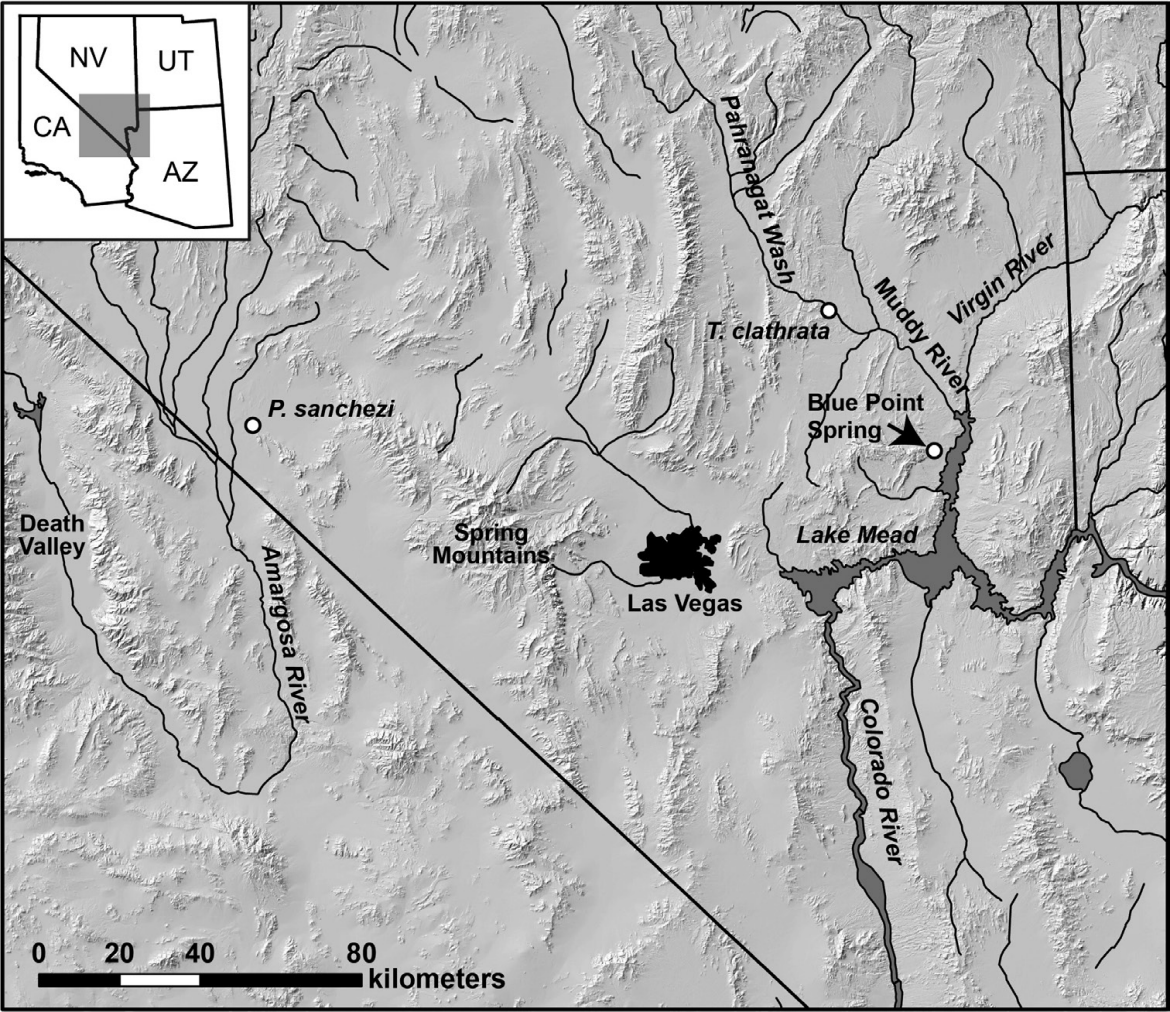
Map showing the location of Blue Point Spring relative to other geographic areas discussed in the text. The collecting localities for specimens of *Pyrgulopsis sanchezi* and *Tryonia clathrata* (sister taxa of Blue Point Spring endemics) used in the molecular phylogenetic analyses are also shown.

The *Assiminea infima* complex is subdivided into a clade that is distributed in the Death Valley region (this lineage also contains a population from the head of the Gulf of California) and a genetically divergent population (Assiminea aff. infima Berry) in Blue Point Spring (Hershler and Liu 2008a), which is located along Lake Mead ca. 150 km to the east-southeast (Fig. 1). Blue Point Spring also contains an endemic species of *Pyrgulopsis* (*Pyrgulopsis coloradensis* Hershler) and a population of *Tryonia* that was previously assigned to widely ranging *Tryonia porrecta* (Hershler 2001), neither of which have been previously studied genetically. Here we analyze newly obtained and previously published DNA sequences to examine the intersection of the biogeographic histories of the three contrasting groups of snails at this isolated spring. We also describe the Blue Point *Tryonia* as a new, endemic species based on molecular and morphologic evidence. Our results reveal a complex historic assembly of the Blue Point Spring snail fauna; and delineate this water body as another micro-hotspot of groundwater-dependent biodiversity in the region, which will assist ongoing efforts to protect and conserve these imperiled ecosystems (Greenwald and Bradley 2008, Abele 2011).

## Methods

Fresh material was collected from Blue Point Spring by RH in May, 2014, and preserved in 90% ethanol for genetic analysis; a portion of the *Tryonia porrecta* sample was relaxed with menthol crystals, fixed in dilute (4%) formalin, and preserved in 70% ethanol for anatomical study. Genomic DNA was extracted from entire snails (Assiminea aff. infima, six specimens; *Pyrgulopsis coloradensis*, four specimens; *Tryonia porrecta*, six specimens) using a CTAB protocol (Bucklin 1992); each specimen was analyzed for mtDNA separately. LCO1490 and HCO2198 (Folmer et al. 1994) were used to amplify a 710 base pair (bp) fragment of the cytochrome *c* oxidase subunit I gene (COI). Amplification conditions and sequencing of amplified polymerase chain reaction product were those of Liu et al. (2003). Sequences were determined for both strands and then edited and aligned using Sequencher^TM^ version 5.0.1. Novel haplotypes were not detected in the newly sequenced specimens of Assiminea aff. infima and thus we did not update our previously published phylogenetic analysis of the *Assiminea infima* complex (Hershler and Liu 2008a). The newly sequenced specimens of Blue Point Spring *Tryonia* were analyzed together with previously published sequences from 30 congeners and closely related *Minckleyella balnearis* Hershler, Liu & Landye (a monotypic genus from northern Mexico), with *Mexipyrgus carranzae* Taylor used to root the phylogenetic tree (per Liu et al. 2001). Given that *Pyrgulopsis* is a large genus containing 139 species (Hershler et al. 2014), most of which have been previously sequenced, we restricted our analysis of the relationships of *Pyrgulopsis coloradensis* to the newly sequenced specimens from Blue Point Spring, and sequences of 18 congeners from adjacent areas (including those that were found to be most similar to the newly obtained haplotypes using a BLAST search) to obtain a readable tree. The phylogenetic tree for this dataset was rooted with *Floridobia winkleyi* (Pilsbry) (per Hershler et al. 2003). One example of each haplotype detected in a given sample was used in the phylogenetic analyses. The new sequences from Blue Point Spring populations were deposited in GenBank (accession numbers KP899916–KP899919).

MrModeltest 2.3 (Nylander 2004) was used to obtain an appropriate substitution model (using the Akaike Information Criterion) and parameter values for the molecular phylogenetic analyses. MRMODELTEST selected GTR + I + G model parameters as the best fit model for both the *Tryonia* and *Pyrgulopsis* datasets. Phylogenetic analyses were performed using four different methodologies—distance, maximum parsimony (MP), maximum likelihood (ML) and Bayesian inference. The distance, MP, and ML analyses were performed using PAUP*4.ob10 (Swofford 2002), and the Bayesian analyses were conducted using MrBayes 3.2.3 (Ronquist and Huelsenbeck 2003). For the distance analyses, GTR distance was used to generate a neighbor-joining (NJ) tree (Saitou and Nei 1987). The MP analyses were conducted with equal weighting, using the heuristic search option with tree bisection reconnection branch-swapping and 100 random additions. The ML analyses were performed using GTR + I + G model. A GTR distance based NJ tree was used as the initial topology for branch-swapping. Node support was evaluated by 10,000 bootstrap pseudo-replicates except for the ML analysis, in which support values were based on 100 replications. For the Bayesian analyses Metropolis-coupled Markov chain Monte Carlo simulations were run with four chains (using the model selected through MrModeltest) for 3,000,000 generations for *Tryonia*, and 2,000,000 generations for *Pyrgulopsis*. Markov chains were sampled at intervals of 10 generations to obtain 300,000 and 200,000 sample points, respectively. We used the default settings for the priors on topologies and the GTR + I + G model parameters selected by MrModeltest as the best fit model for both analyses. At the end of the analyses, the average standard deviation of split frequencies was less than 0.01 (0.0036 and 0.0033, respectively) and the Potential Scale Reduction Factor (PSRF) was 1, indicating that the runs had reached convergence. The sampled trees with branch lengths were used to generate a 50% majority rule consensus tree, with the first 25% of the samples removed to ensure that the chain sampled a stationary portion.

Genetic distances within and between samples were calculated using MEGA6 (Tamura et al. 2013), with standard errors estimated by 1,000 bootstrap replications with pairwise deletion of missing data. Since MEGA does not contain the GTR model that was selected by MRMODELTEST, we used the maximum composite likelihood distance, which is the nearest model. Tajima relative rate tests of local clock-like behavior (Tajima 1993) were performed using MEGA6. The posterior Bayes factor was used to test a global clock assumption (MRBAYES 3.2.3).

Large, adult females were used for shell measurements. The total number of shell whorls (WH) was counted for each specimen; and the height and width of the entire shell (SH, SW), body whorl (HBW, WBW), and aperture (AH, AW) were measured from camera lucida outline drawings using a digitizing pad (see Hershler 1989). In addition, three ratios were generated from the raw data (SW/SH, HBW/SH, AH/SH). Descriptive statistics were generated using Systat for Windows 11.00.01 (SSI 2004). Sexual dimorphism in shells, which is commonly observed in *Tryonia* species (Taylor 1987), could not be quantified owing to the small sample size. Variation in the number of cusps on the radular teeth (*n* = 5) was assessed using the method of Hershler et al. (2007a). Descriptive terminology follows that of Taylor (1987) and Hershler (2001). The brief taxonomic description of the new species focuses on diagnostic features of external morphology. Types and other voucher material were deposited in the Smithsonian Institution’s National Museum of Natural History (USNM) collection.

## Results

**Figure 2.**
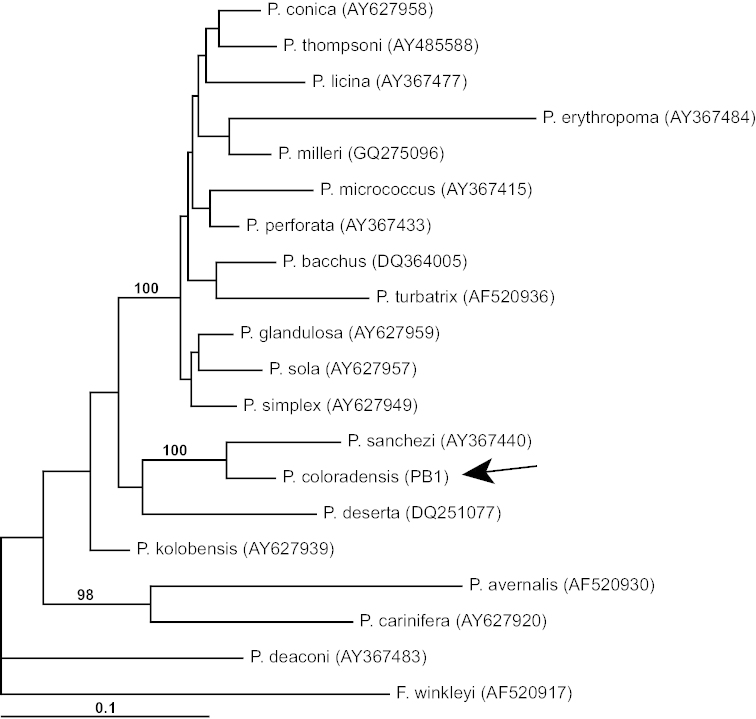
Bayesian tree based on COI data delineating the phylogenetic relationships of *Pyrgulopsis coloradensis* (sequence identified by arrow). Posterior probabilities for nodes are indicated when >95%. GenBank accession numbers for haplotypes are given in parentheses.

The phylogenetic analyses congruently depicted a sister relationship between *Pyrgulopsis coloradensis* and *Pyrgulopsis sanchezi* Hershler, Liu & Bradford, which is distributed in the Death Valley area (Fig. 1). This relationship was strongly supported (1.0 posterior probability) only in the Bayesian analysis (the Bayesian tree is shown in Fig. 2). This clade in turn was depicted as sister to *Pyrgulopsis deserta* (Pilsbry) (distributed along the Colorado River upflow from Blue Point Spring), albeit without support (0.70). The four sequenced specimens of *Pyrgulopsis coloradensis* shared the same haplotype which differed from sequences of the other congeners included in the analysis by 4.5–11.4%.

**Figure 3.**
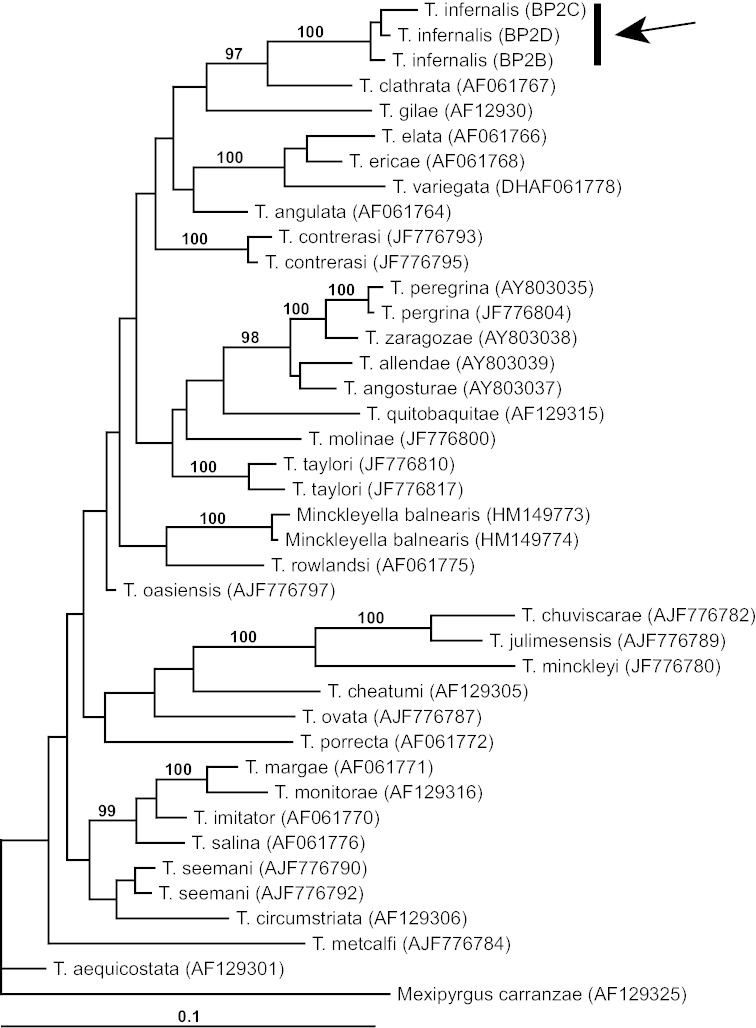
Bayesian tree based on COI data delineating the phylogenetic relationships of the Blue Point Spring *Tryonia* (lineage highlighted by arrow). Posterior probabilities for nodes are indicated when >95%. GenBank accession numbers for haplotypes are given in parentheses.

The phylogenetic analyses of the *Tryonia* dataset congruently delineated a well-supported sister relationship between Blue Point Spring population and *Tryonia clathrata*, which is also distributed in the lower Colorado River basin (Fig. 1). The Bayesian tree is shown in Fig. 3. (Note that the haplotype detected in near topotypes of *Tryonia porrecta* was positioned in another portion of the tree.) The depicted sister relationship between this clade and *Tryonia gilae* Taylor (which is also distributed in the lower Colorado River basin) was not well supported. Three haplotypes (BPB-D) differing by 3–7 bps were detected in the six sequenced specimens of the Blue Point Spring *Tryonia*. The average divergence between these haplotypes and those of the other congeners included in the analysis was 3.9–9.0%. The Blue Point Spring population of *Tryonia* is morphologically diagnosable (as detailed below) in addition to being phylogenetically independent and substantially divergent genetically. We describe this distinct evolutionary lineage as a new species below.

The eight sequenced specimens of Assiminea aff. infima shared the same haplotype which differed from sequences of the other members of the *Assiminea infima* complex by 2.6 +/- 0.5%.

Tajima’s relative rate test did not reject clocklike behavior for the datasets of interest. The posterior Bayes factor also strongly favored the molecular clock model, indicating that the application of a molecular clock is appropriate for these data.

## Systematic description

### Family Cochliopidae Tryon, 1866 Genus *Tryonia* Stimpson, 1865

#### Tryonia infernalis

Taxon classificationAnimaliaLittorinimorphaCochliopidae

Hershler, Liu, & Simpson sp. n.

http://zoobank.org/F7DD4C5E-E128-48AC-BAAE-866B6980C869

Tryonia infernalis Undescribed [*Fontelicella* and] *Tryonia* species.—Williams et al. 1985: 32.
Tryonia porrecta .—Hershler 1999: 335.

##### Types.

USNM 883884 (a dry shell), Blue Point Spring, just below source, Clark County, Nevada, 36.3894°N, 114.4329°W, 24 July 1988, R. Hershler. Paratypes (ca. 200 dry shell and alcohol preserved specimens), USNM 1266143 (from same lot).

##### Referred material.

NEVADA. *Clark County*: USNM 883248 (coll. James J. Landye, 17-XII-1992), USNM 1098627 (coll. Donald W. Sada, 6-XII-2006), USNM 1146345 (coll. Andrew K. Schwaneflugel, 29-V-2008), USNM 1146420 (coll. DWS, 11-XII-2009), USNM 1248362 (coll. RH, 5–15–2014), USNM 854844 (coll. Saxon Sharpe, no date), Blue Point Spring.

##### Diagnosis.

Shell medium-sized, conic to turriform; penis having two distal papillae on the inner edge and a single basal papilla both on the inner and outer edges. Readily distinguished from geographically proximal and closely related *Tryonia clathrata* by its smaller size, weaker shell sculpture, and smaller number of papillae on the inner edge of the penis. Differentiated from *Tryonia gilae* (also distributed in the lower Colorado River basin) by its more convex teleoconch whorls, lateral expansion of distal bulb of penis, and in having a basal papilla on the inner edge of the penis. Differs from *Tryonia porrecta*, with which it was previously confused, by its smaller size, consistently weak shell sculpture, and much greater frequency of males.

##### Description.

**Figure 4.**
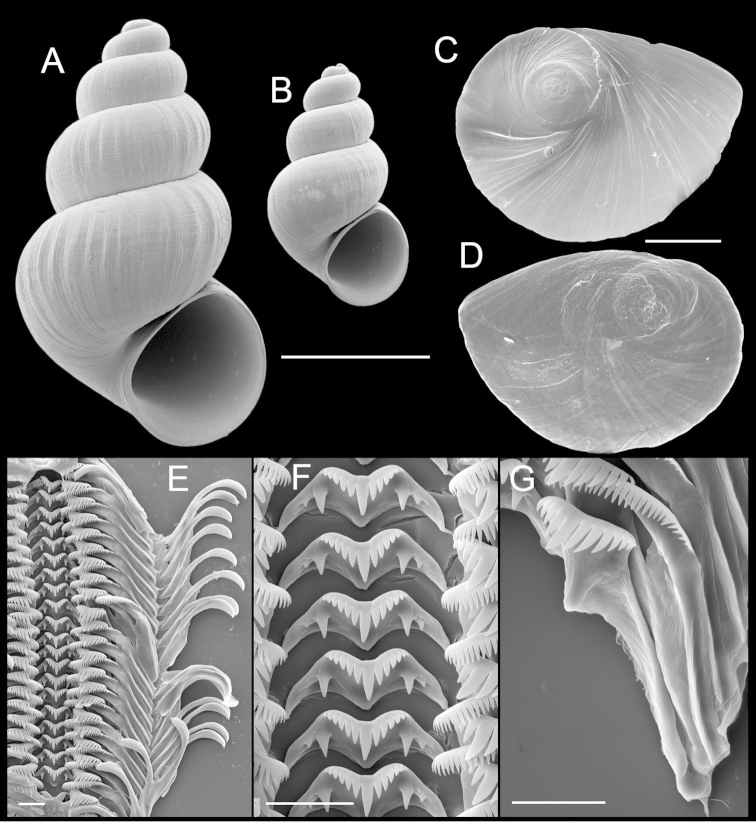
Shells, opercula and radula, *Tryonia infernalis* sp. n. **A** Holotype, USNM 883884 **B** Male shell, USNM 1266143 **C, D** Opercula (outer, inner sides), USNM 1266143 **E** Portion of radular ribbon, USNM 1266143 **F** Central teeth, USNM 1266143 **G** Lateral and inner marginal teeth, USNM 1266143. Scale bars **A–B:** 1.0 mm; **C, D:** 200 µm; **E–G:** 10 µm.

Shell (Fig. 4A–B) up to 2.8 mm tall, large females having 5.00–5.75 whorls, spire height 100–133% width of shell, male shells smaller than those of females. Teleoconch whorls highly convex, evenly rounded. Aperture ovate, weakly angled adapically. Parietal lip complete, adnate, umbilicus narrow. Outer lip orthocline or prosocline, sometimes weakly sinuate. Sculpture of strong growth lines and a few weak spiral threads. Periostracum light brown. Shell parameters for a series of paratypes are given in Table 1.

**Table 1.** Shell parameters for *Tryonia infernalis*. Measurements are in mm.

	WH	SH	SW	HBW	WBW	AH	AW	SW/SH	HBW/SH	AH/SH
Holotype, USNM 883884										
	5.75	3.09	1.78	1.87	1.56	1.13	1.00	0.58	0.604	0.36
Paratypes, USNM 1266143 (*n* = 9)										
Mean	5.33	2.61	1.41	1.60	1.25	0.95	0.80	0.54	0.61	0.37
S.D.	0.28	0.15	0.06	0.07	0.06	0.04	0.04	0.03	0.03	0.02
Range	5.00–5.75	2.41–2.82	1.33–1.54	1.46–1.71	1.13–1.34	0.91–1.02	0.75–0.86	0.49–0.59	0.58–0.65	0.34–0.39

Inner and outer sides of operculum smooth (Fig. 4C–D). Radula (Fig. 4E–G): dorsal edge of central teeth concave, basal tongue V-shaped, median cusps elongate, distally pointed, lateral cusps four–six, basal cusps one–two, usually two (innermost larger; Fig. 4F). Lateral teeth having three–five cusps on inner and five–seven cusps on outer side, length of outer wing about 200% width of cutting edge, central cusp pointed (Fig. 4G). Inner marginal teeth with 24–34 cusps, outer marginal teeth with 27–38 cusps. Radula data are from USNM 1266143.

**Figure 5.**
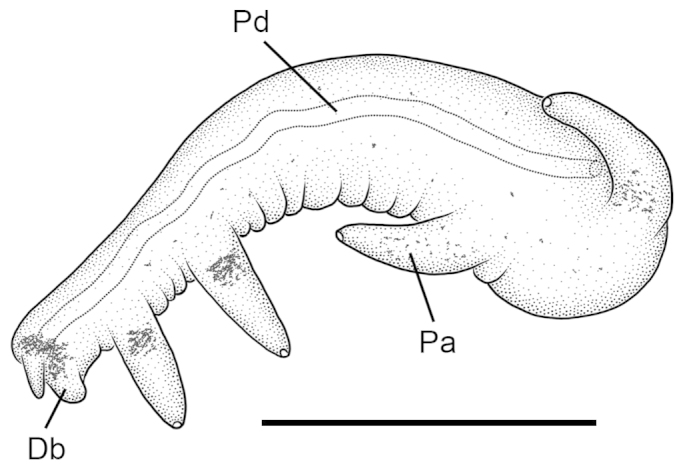
Penis (dorsal surface), *Tryonia infernalis*, USNM 1248362. Scale bar: 500 µm. **Db** distal bulb **Pa** distal papilla **Pd** penial duct.

Animal darkly pigmented. Penis (Fig. 5) having two distal and one basal papillae on inner edge and one basal papilla on outer edge (29 of 30 specimens); one specimen differed in having a single distal papilla along the inner edge. Distal bulb of penis expanded laterally on inner side, black; stylet small. Penial duct weakly undulating along most of length. Penial data are from USNM 1248362.

##### Etymology.

The specific epithet (infernalis) is a Latin adjective meaning hellish, and refers to the Valley of Fire, which is closely proximal to the type locality.

##### Distribution and habitat.

**Figure 6.**
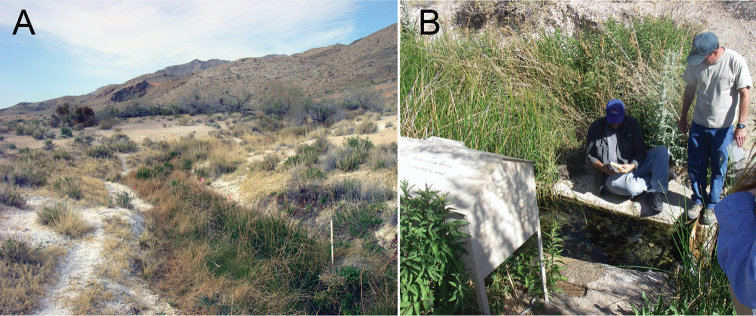
Photographs of Blue Point Spring. **A** Outflow channel; spring originates below one of the mesquite trees in the upper right (photograph taken on 24/III/2009) **B** Ponded area where *Tryonia infernalis* occurs abundantly; the USGS gage house is in the lower left (15/V/2014).

*Tryonia infernalis* is known only from its type locality, a thermal (ca. 30 °C) rheocrene (discharging ca. 0.55 l/s; USGS 2007) whose outflow forms a narrow (ca. 0.3 m) stream (Fig. 6A). *Tryonia infernalis* is restricted to the upper 10 m of the spring run where it lives on silt and rocks. This species was considered to be extinct in 2002 following a series of unsuccessful searches, however it was subsequently “rediscovered” in a small, ponded reach above a weir plate associated with a USGS gaging station (Fig. 6B) in 2007 (Sada and Jacobs 2008; also see CCDCP 2002) and currently is abundant at this site (RH, personal observation).

##### Remarks.

The reproductive anatomy of several females was studied to confirm that this species belongs to *Tryonia* as currently defined (Hershler 2001).

## Discussion

Small assemblages of locally endemic spring-dwelling invertebrates are scattered throughout arid western North America (Williams et al. 1985, Shepard 1993, Myers and Resh 1999). Although the biogeographic history of (some of) these taxa has been studied at spatial scales ranging from local watersheds (e.g., Hershler et al. 2007b) to major hydrographic basins (e.g., Witt et al. 2008) to the entire region (Liu and Hershler 2005), the origins of the endemic faunas of individual springs have been little investigated. The molecular phylogenetic evidence clearly points to a minimally dual origin of the Blue Point Spring fauna—*Tryonia infernalis* is sister to a geographically close species from the lower Colorado River basin whereas *Pyrgulopsis coloradensis* and the Blue Point Spring *Assiminea* are most closely related to taxa in the Death Valley region well to the west. (Note that Blue Point Spring harbors a divergent lineage of *Hyalella* amphipods that also is closely related to populations in the Death Valley region; Witt et al. 2006, provisional species HaPS11).

The use of a molecular clock to estimate divergence times is wrought with difficulties and is further complicated in this case by the absence of locally derived calibrations for *Assiminea* and *Tryonia*. Nevertheless, roughly calculated values provide useful insight into the biogeographic history of the Blue Point Spring fauna (note that we performed Bayes factor and Tajima’s rate tests, both of which suggested that the assumption of a molecular clock is valid). Based on mtCOI clock calibrations of 1.83% per million years. for European Hydrobiidae (Wilke 2003) and 1.62% per m.y. for *Pyrgulopsis* (Hershler and Liu 2008b), the estimated divergence times of the snail populations in Blue Point Spring ranged from 1.42–2.78 Ma (Table 2). Although the age of Blue Point Spring is not known with certainty, middle to lower Pleistocene (≤2.6 Ma) spring deposits (Beard et al. 2007, map unit Q2s) provide the earliest record of local groundwater discharge. Thus, the endemic lineages may be roughly with the same age as or slightly younger than Blue Point Spring. Lake Mead and the Death Valley region are separated by the intervening, north-south trending Spring Mountains (Fig. 1) and there is no record of a prior drainage connection between these areas during the Neogene; thus it would seem likely that *Assiminea* and *Pyrgulopsis* were transported to Blue Point Spring on waterbirds. The molecular data presented here suggests that these two groups may have colonized Blue Point Spring at different times during the Pleistocene (Table 2). The sister taxon of *Tryonia infernalis* (*Tryonia clathrata*) is distributed in the White River Valley, which drains into Lake Mead (via the Muddy River) a few kilometers upflow from Blue Point Spring (Fig. 1). The split between these geographically close lineages could have been a product of vicariance (e.g., severance of a thermal stream connection per Hershler et al. 1999a) or dispersal of birds (per Wesselingh et al. 1999). Whereas only a single haplotype was detected for specimens of both *Pyrgulopsis coloradensis* and the Blue Point Spring *Assiminea*, three well differentiated haplotypes (mean divergence, 0.6%) were observed in *Tryonia infernalis*, suggesting a possibly longer history of *in-situ* diversification. Our findings imply a relatively complex assembly of the Blue Point Spring snail fauna. The mixture of a locally derived element that may have a relatively long history of diversification within the spring (*Tryonia infernalis*), and lineages that appear to have colonized this water body more recently (with no subsequent differentiation) follows a common pattern of community assembly (Emerson and Gillespie 2008).

**Table 2.** COI sequence divergence and estimated ages of Blue Point Spring snail lineages based on two clock calibrations.

Lineage	Per cent sequence divergence (sister taxon)	Estimated age (m.y.)	
		1.83%/m.y. calibration^1^	1.62%/m.y. calibration^2^
Assiminea aff. infima	2.6 (other members of *Assiminea infima* complex)	1.42	1.60
*Pyrgulopsis coloradensis*	4.5 (*Pyrgulopsis sanchezi*)	2.46	2.78
*Tryonia infernalis*	3.9 (*Tryonia clathrata*)	2.13	2.41

1 Wilke (2003)

2 Hershler and Liu (2008b)

The recognition of *Tryonia infernalis* as a distinct, endemic species further highlights Blue Point Spring as a micro-hotspot of locally endemic aquatic biodiversity in Nevada. The Blue Point Spring *Assiminea* is probably a distinct species as well, but a formal taxonomic treatment is deferred pending completion of an ongoing revision of the *Assiminea infima* complex (Hershler and Liu in preparation). Although this tiny aquatic ecosystem is on lands administered by the National Park Service (Lake Mead National Recreation Area), there may be a need for additional protection and conservation measures. The spring is located alongside a paved highway and public access is further facilitated by a small parking area near the lower end of the spring run. There is no fencing around the spring (or its run) and thus it is vulnerable to disturbance from foot traffic and other recreational activities. [We note in this context that Blue Point Spring harbors one of the few remaining populations of the relict leopard frog (*Rana onca* Cope), which requires open habitat maintained by ungulate grazing and thus may be negatively impacted by fencing (Bradford et al. 2004).] The spring run appears to have been “channelized” at one time in the past, which likely resulted in a reduction of the riparian habitat utilized by *Assiminea* (Landye 1973). The snail fauna may be further jeopardized by a suite of exotic fishes that were introduced through the use of the spring as an aquarium-fish rearing establishment (until the mid-1950’s) or by aquarium release (Deacon et al. 1964); the convict cichlid (*Amatitlania nigrofasciata* [Günther]), which was discovered in the spring in the 1990’s, may pose an especially serious threat owing to its omnivorous feeding habitats (Sada and Jacobs 2008). The red-rimmed melania (*Melanoides tuberculata* [Müller]), an invasive gastropod whose abundance appears to be negatively correlated with that of native snails in western springs based on anecdotal evidence, has also been introduced to the spring (Landye 1973).

## Supplementary Material

XML Treatment for Tryonia infernalis
